# Ovarian toxicity of carboplatin and paclitaxel in mouse carriers of mutation in *BRIP1* tumor suppressor gene

**DOI:** 10.1038/s41598-022-05357-x

**Published:** 2022-02-01

**Authors:** E. Ntemou, P. Diaz Vidal, C. Alexandri, G. Van den Steen, M. Lambertini, I. Demeestere

**Affiliations:** 1 Research Laboratory on Human Reproduction, Université Libre de Bruxelles, Brussels, Belgium; 2 Department of Internal Medicine and Medical Specialties (DiMI), School of Medicine, University of Genova, Genoa, Italy; 3 Department of Medical Oncology, UOC Clinica di Oncologia Medica, IRCCS Ospedale Policlinico San Martino, Genoa, Italy; 4 Obstetrics and Gynaecology Department, Erasme Hospital, Brussels, Belgium

**Keywords:** Breast cancer, Gonadal disorders, Chemotherapy

## Abstract

More than 10% of women diagnosed with breast cancer during reproductive age carry hereditary germline pathogenic variants in high-penetrance *BRCA *genes or in others genes involved in DNA repair mechanisms such as *PALB2*, *BRIP* or *ATM*. Anticancer treatments may have an additional negative impact on the ovarian reserve and subsequently on the fertility of young patients carrying such mutations. Recently, the combination of carboplatin and paclitaxel is being recommended to these *BRCA*-mutated patients as neoadjuvant therapy. However, the impact on the ovary is unknown. Here, we investigated their effect of on the ovarian reserve using mice carriers of BRCA1-interacting protein C-terminal helicase-1 (*BRIP1*) mutation that plays an important role in *BRCA1*-dependent DNA repair. Results revealed that the administration of carboplatin or paclitaxel did not affect the ovarian reserve although increased DNA double-strand breaks were observed with carboplatin alone. Co-administration of carboplatin and paclitaxel resulted in a significant reduction of the ovarian reserve leading to a lower IVF performance, and an activation of the PI3K-Pten pathway, irrespective of the genetic background. This study suggests that co-administration of carboplatin and paclitaxel induces cumulative ovarian damage and infertility but a heterozygote genetic predisposition for DNA damage related to *BRCA1* gene function does not increase this risk.

## Introduction

Breast cancer is the most common cancer type in young adults worldwide, with the breast cancer global incidence in 20–39-year-old females at 17.0 per 100,000 people per year^1^. Germline pathogenic variants in the breast cancer susceptibility genes *BRCA1* and *BRCA2* account for about 12% of the cases^2^. Other germline mutations in genes involved in DNA repair such as *RAD51*, *PALB2* or *BRIP* confer cancer susceptibility, highlighting their crucial role in maintaining genome integrity.

For these patients, fertility issues may be of particular concern as previous studies suggested a possible negative impact of *BRCA* pathogenic variants on ovarian reserve and fertility^3,4^. BRCA is known to be involved in homologous DNA recombination, and plays an essential role in DNA double-strand breaks (DSBs) repair protecting the integrity of the genome in proliferating cells. Pathogenic variants in *BRCA* genes, and especially in *BRCA1*, result in deficient homologous-recombination DNA repair and chromosomal instability. Besides the high risk of developing breast and ovarian cancers, deficient DNA repair in *BRCA*-mutated patients may leave oocytes more prone to DNA damage and aging resulting in diminished ovarian reserve^5,6^.

Major advances in anticancer therapies have contributed to increase the survival rate over the last years. However, these treatments may have some long-term side effects including a negative impact on ovarian germ cells that may lead to premature ovarian insufficiency (POI) and subsequent infertility^7,8^. The negative impact on the ovarian reserve could be even more of concern in *BRCA*-mutated breast cancer patients. The investigation of the gonadotoxic burden of new anticancer therapies is a research priority, especially for patients harbouring *BRCA* pathogenic variants^9,10^, considering that this knowledge is a prerequisite to offer adequate fertility preservation counselling.

The association of carboplatin and paclitaxel has been recently implemented in the treatment of young *BRCA*-mutated breast cancer patients^7^. Carboplatin, developed in the early 1980s, is a second-generation platinum compound that is less toxic than cisplatin with comparable antitumor activity^11^. So far, carboplatin has been used for the treatment of various cancers, including advanced ovarian cancer of epithelial origin, lung cancer, and less extensively in testicular cancer, head and neck cancer, leukaemia, brain and breast cancer^12,13^. Carboplatin acts by generating lesions in the DNA through the formation of adducts with platinum, thereby inhibiting replication and transcription and inducing cell death^14^. Carboplatin, similar to cisplatin, is characterised as a gonadotoxic drug and may be particularly gonadotoxic for *BRCA*-mutated breast cancer patients considering their DNA repair deficiency^15,16^. Paclitaxel is a widely used chemotherapeutic drug that belongs to taxanes and is used to treat lung, cervical and breast cancer^17^. Its mechanism of action is based on binding to tubulin, the protein component of microtubules, and stabilizing the microtubules. By disturbing the normal dynamic reorganization of the microtubule network, cells remain in the M phase of the cell cycle and cell division is inhibited leading to cell death^18^. Studies on the gonadotoxicity of paclitaxel have been controversial^19^. Moreover, the gonadotoxicity of combined regimen including both carboplatin and paclitaxel is unknown, particularly in the cases of mutations involved in DNA repair mechanisms such as *BRCA1*, *BRIP*, *RADF51*.

In the present study, we aimed to evaluate the gonadotoxic effect of carboplatin and paclitaxel alone or in combination and to further investigate whether a defect in molecular pathways involved in DNA repair mechanisms can increase ovarian damage. Beside *BRCA* genes, at least 15 other genes, linked to Fanconi anaemia pathway, are involved in the DSB repair pathway^21^. In order to address more specifically the role of the DNA repair mechanism in treatment-induced ovarian damage, we selected a mouse strain with a mutation involved in *BRCA1*-related DNA repair mechanisms: the CRISPR/cas9 mutant of the *BRCA1* interacting protein C-terminal helicase 1 (*Brip1*) gene (also known as *BACH1* or *FANCJ)*^22^*.* In contrast with the homozygote germline *BRCA1* knock-out model^20^, both heterozygote and homozygote *Brip1* mutants are viable. *Brip1*, encodes a DNA-dependent ATPase which interacts directly with the C-terminal BRCT repeat of BRCA1. The exact function of Brip1 is still not clear but it seems to be involved in the processing of intermediate DNA structures during repair or by providing a scaffold for other homologous recombination proteins^23^. Another model suggests that BRCA1 regulates the BRIP1 helicase activity to coordinate the timely displacement of Rad51 from DNA strands to complete the HR process^24^. Therefore, the interaction between BRCA1 and BRIP1 is important for promoting error-free DSB repair and maintaining chromosomal integrity^25^. Additionally, heterozygous mutation of *Brip1* predisposes to early onset breast cancer supporting its role in hereditary breast cancer development and *BRCA1*-related DNA repair mechanisms^26^.

## Results

### Co-administration of carboplatin and paclitaxel reduces the ovary to body weight ratio in both mutant carriers and wild-type mice

**Figure 1:**
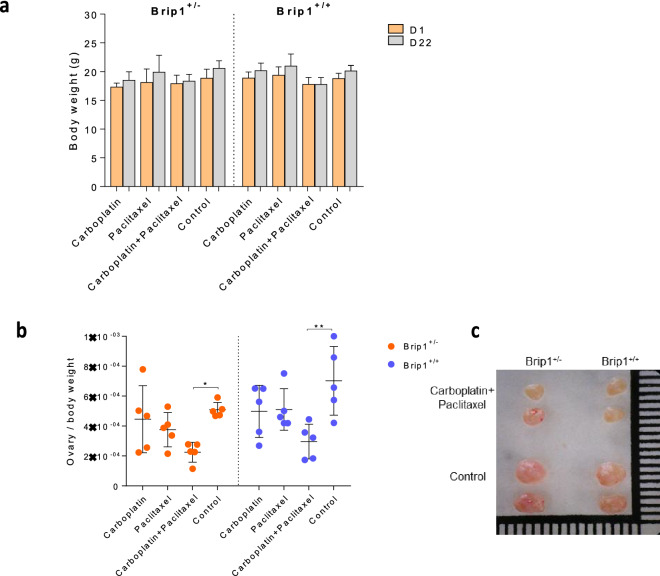
(**a**) Body weight of *Brip1*^+*/−*^* and Brip1*^+*/*+^ mice between the first (D1) and the last (D22) day of the experiments. Data are presented as mean ± SD; n = 5/treatment/genotype. (**b**) Ovary to body weight ratio of *Brip1*^+*/−*^* and Brip1*^+*/*+^ mice at the end of the treatment. Data are presented as mean ± SD; n = 5/treatment/genotype; *p < 0.05, **p < 0.01. (**c**) Image illustrating the differences in the size of the ovaries of *Brip1*^+*/−*^* and Brip1*^+*/*+^ mice treated with the combination of carboplatin and paclitaxel or with NaCl 0.9% (control group) at the end of the treatment.

*Brip1*^*−/−*^*, Brip1*^+*/−*^* and Brip1*^+*/*+^ mice were treated with carboplatin, paclitaxel, the combination of carboplatin and paclitaxel (combined treatment group) or with serum alone (control), according to the described protocol for three weeks. *Brip1*^*−*/*−*^ mice did not survive the co-administration of carboplatin and paclitaxel and therefore were excluded from the study. The mean weight of mice did not change significantly between the first and last day of the experiments for any of the treatment groups (Fig. 1a). However, co-administration of carboplatin and paclitaxel resulted in lower ovary to body weight ratio in both *Brip1*^+/*−*^ (p < 0.05) and *Brip1*^+/+^ (p < 0.01) mice at the time of sacrifice (Fig. 1b,c).

### Co-administration of carboplatin and paclitaxel results in reduced ovarian reserve in both mutant carriers and wild-type mice

**Figure 2:**
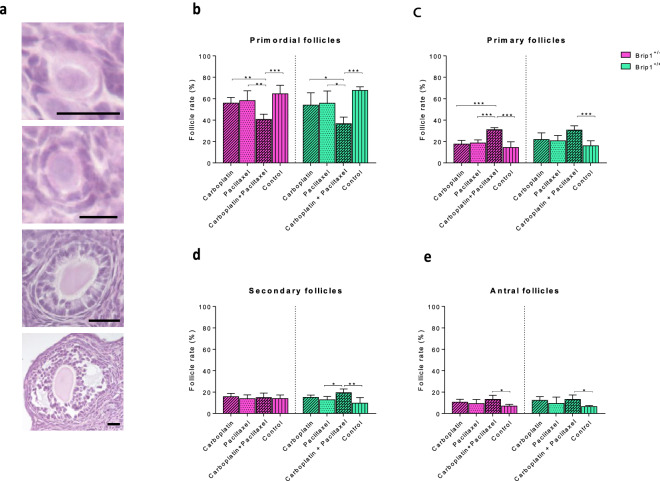
Effect of the treatment with carboplatin (50 mg/kg, once a week), paclitaxel (10 mg/kg, twice a week) and the combination of carboplatin and paclitaxel (50 mg/kg, once a week and 10 mg/kg, twice a week) on the percentage of follicles in different developmental stages of *Brip1*^+*/−*^* and Brip1*^+*/*+^ mice compared to the control group (NaCl 0.9%) at the end of the three-week treatment protocol. (**a**) Representative micrographs from haematoxylin and eosin-stained follicles at different developmental stages. From top to bottom, primordial, primary, secondary and antral follicles (scale bar = 20 μm). Effect of the treatment on the population of primordial (**b**), primary (**c**), secondary (**d**) and antral follicles (**e**). Results are expressed as the percentage of the total number of healthy follicles per ovary. Data are presented as mean ± SD; n = 5/treatment/genotype; *p < 0.05, **p < 0.01, ***p < 0.001.

To evaluate the effect of the drugs on the follicular reserve, the rates of follicles (primordial, primary, secondary, and antral) to the total number of follicles were calculated first. Co-administration of carboplatin and paclitaxel to *Brip1*^+/*−*^ mice resulted in a decline in the percentage of primordial follicles compared to the control (− 37%; p < 0.001), carboplatin (− 27%; p < 0.01) and paclitaxel (− 30%; p < 0.01) groups (Fig. 2a,b). In addition, the percentage of primary follicles was increased compared to the control group (+ 54%; p < 0.001) (Fig. 2c). Similar increase was observed in the percentage of antral follicles (+ 47%; p < 0.05) (Fig. 2e). The results were similar to the results obtained using *Brip1*^+/+^ mice, as a reduced percentage of primordial follicles was also observed after co-administration of carboplatin and paclitaxel compared to the control (− 46%; p < 0.001), carboplatin (− 32%; p < 0.05) and paclitaxel (− 34%; p < 0.05) groups, while the percentages of primary and antral follicles were increased (+ 51%; p < 0.05 and + 48%; p < 0.001; respectively). The administration of carboplatin or paclitaxel alone to both *Brip1*^+/*−*^ and *Brip1*^+/+^ mice did not affect the ovarian reserve (Fig. 2b–e).

**Figure 3:**
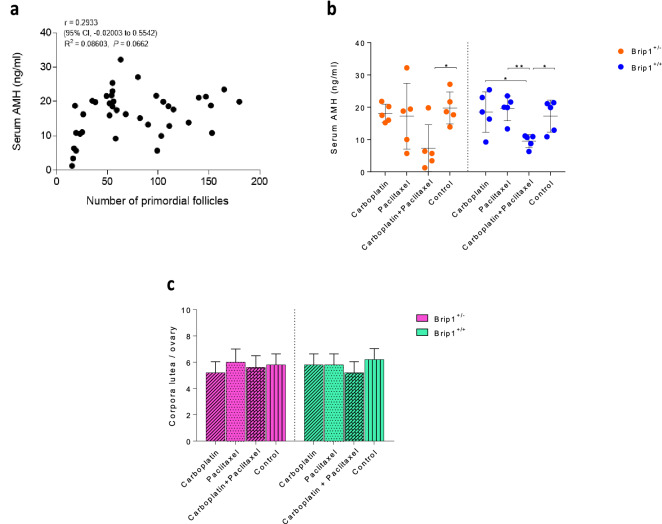
(**a**) Correlation between the AMH serum concentration and the number of primordial follicles in the ovary from all mice used in the study. The graph indicates a trend of the two variables to increase or decrease together. (**b**) AMH serum concentrations of treated *Brip1*^+*/−*^* and Brip1*^+*/*+^ mice quantified by electrochemiluminescence immunoassay at the end of the treatment. (**c**) Total number of corpora lutea counted per ovary. Data are presented as mean ± SD; n = 5/treatment/genotype; *p < 0.05, **p < 0.01.

To confirm the reduced ovarian reserve, anti-müllerian hormone (AMH) levels in the blood serum of mice was assessed at the end of the treatment. Serum AMH levels were significantly decreased following co-administration of carboplatin and paclitaxel in both *Brip1*^+/*−*^ (p < 0.05) and *Brip1*^+/+^ (p < 0.05) mice. As expected, correlation analysis confirmed that the two variables, AMH levels and number of primordial follicles increase or decrease together but statistical significance was not reached (Fig. 3a). Co-administration of carboplatin and paclitaxel in *Brip1*^+/*−*^ or *Brip1*^+/+^ mice induced a decrease in the AMH level reaching significance compared to control, while it did not affect ovulation as indicated by the number of corpora lutea per ovary observed at the end of the treatment (Fig. 3b,c). However, the possible long-term effect on oestrus cycles should be further evaluated after long-term follow-up.

### Co-administration of carboplatin and paclitaxel induces follicular activation in both mutant carriers and wild-type mice

**Figure 4:**
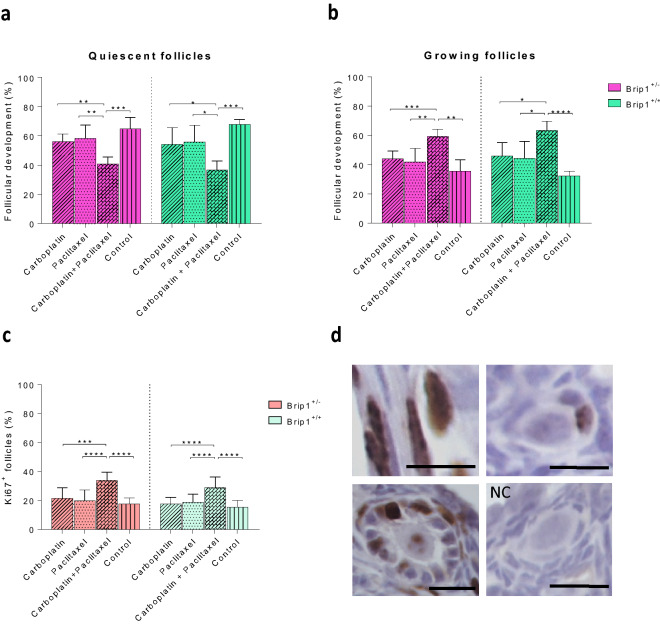
Evaluation of the treatment on follicular activation and development. (**a**) Quiescent follicle (primordial stage) rates and (**b**) growing follicle (primary, secondary and antral stages) rates. (**c**) Proliferative rates of follicles following the treatment. Follicles with at least one granulosa cell stained were counted as positive and reported to the total number of follicles. (**d**) Representative micrographs of Ki67 positive stained follicles. NC, negative control (scale bar 20 μm). Data are presented as mean ± SD; n = 5/treatment/genotype; *p < 0.05, **p < 0.01, ***p < 0.001, ****p < 0.0001.

To quantify follicular activation, the quiescent (primordial) and growing (primary, secondary and antral) follicle rates were compared. Following co-administration of carboplatin and paclitaxel, the quiescent follicle rate was significantly reduced in both *Brip1*^+/*−*^ (p < 0.001) and *Brip1*^+/+^ (p < 0.001) mice compared to control. The combined treatment group exhibited an increased rate of growing follicles (p < 0.01 and p < 0.0001, respectively) indicating drug-induced follicular activation (Fig. 4a,b). Proliferation rate was evaluated by Ki-67 immunostaining. All secondary and antral follicles were positively stained in all treatment groups. Thus, only primordial and primary follicles were quantified based on the presence of one or more Ki67-positive granulosa cells. The percentage of proliferative follicles was significantly increased after co-administration of carboplatin and paclitaxel in both *Brip1*^+/*−*^ (p < 0.0001) and *Brip1*^+/+^ (p < 0.0001) groups compared to the control (Fig. 4c,d).

**Figure 5:**
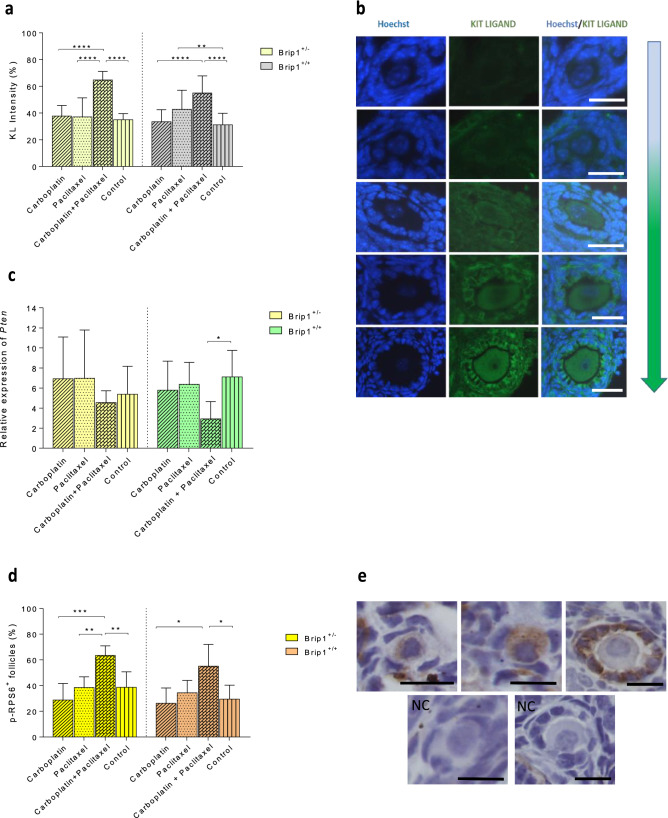
Follicular activation assessment following co-administration of carboplatin and paclitaxel to *Brip1*^+/*−*^ and *Brip1*^+/+^ mice. (**a**) Intensity of KIT LIGAND based on the signal of 20 randomly selected primordial and primary follicles per ovary (scale bar = 20 μm). (**b**) Illustration of the increasing KIT LIGAND staining intensity in primordial and primary follicles (scale bar = 20 μm). (**c**) Expression levels of *Pten* involved in follicular activation in the ovaries of *Brip1*^+/*−*^ and *Brip1*^+/+^ mice. (**d**) Percentage of p-RPS6 positive follicles. (**e**) Representative micrographs of p-RPS6-positive primary and primordial follicles. NC, negative control; scale bar 20 μm. Data are presented as mean ± SD; n = 5/treatment/genotype; *p < 0.05, **p < 0.01, ***p < 0.001, ****p < 0.0001.

Elevated KIT LIGAND (KL) intensity in the primordial and primary follicles of the combined treatment group confirmed the follicular growth in both *Brip1*^+/*−*^ (p < 0.0001) and *Brip1*^+/+^ (p < 0.0001) mice (Fig. 5a,b). Moreover, the expression of *Pten* gene was downregulated in the ovaries following co-administration of carboplatin and paclitaxel suggesting the activation of the PI3K/AKT/mTOR pathway although the difference was not statistically significant for *Brip1*^+/*−*^ mice (Fig. 5c).

To confirm this result, immunostaining for phosphorylated 40S ribosomal protein S6 (p-RPS6, one of the effectors of the PI3K pathway) was performed. All secondary and antral follicles were stained positive in all treatment groups and controls. The percentage of p-RPS6-positive follicles (primordial and primary) was increased in both *Brip1*^+/*−*^ and *Brip1*^+/+^ mice after co-treatment (Fig. 5d,e).

### Co-administration of carboplatin and paclitaxel induces DNA double-strand breaks (DSB) in primordial follicles in both mutant and wild-type mice

**Figure 6:**
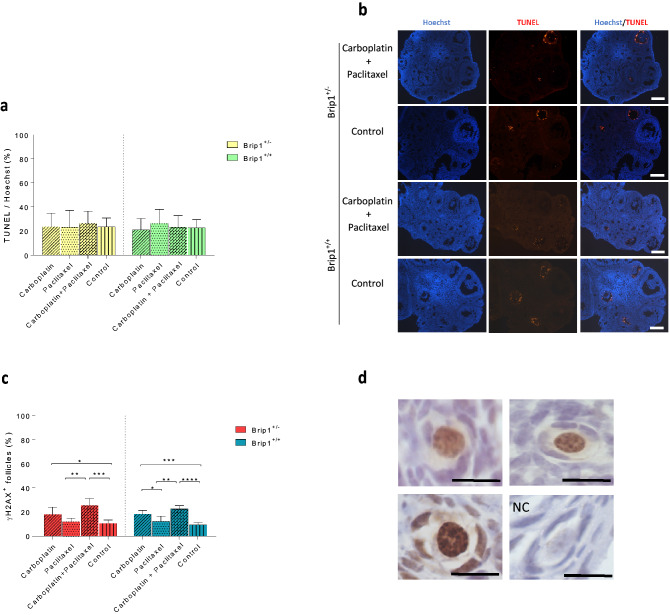
Evaluation of apoptosis and DNA damage in the ovaries of *Brip1*^+/*−*^ and *Brip1*^+/+^ mice. (**a**) Quantification of the degree of global apoptosis in the ovaries of treated mice. (**b**) Representative micrographs of nuclear labelling with Hoechst (blue) and TUNEL-positive (red) apoptotic cells from ovarian sections from mice treated with the combination of carboplatin and paclitaxel and the control group (scale bar = 200 μm). (**c**) Percentage of γH2AX-positive primordial follicles. (**d**) Representative micrographs of γH2AX-positive primordial follicles. NC, negative control; scale bar = 20 μm. Data are presented as mean ± SD; n = 5/treatment/genotype; *p < 0.05, **p < 0.01, ***p < 0.001, ****p < 0.0001.

To assess the possible damage of the drugs on the mouse ovaries, apoptosis was evaluated in all groups by TUNEL assay. The degree of global apoptosis did not differ between treatment groups and control or between *Brip1*^+/*−*^ and *Brip1*^+/+^ mice with the TUNEL-positive cells mostly located in antral follicles (Fig. 6a,b). DNA damage was further investigated by the presence of phosphorylated H2AX (γH2AX), a well-defined biomarker for DSB, in the nucleus of primordial follicles. The percentage of follicles with DSB was increased in the ovaries of *Brip1*^+/*−*^ (p < 0.001) and *Brip1*^+/+^ (p < 0.001) mice treated with the combination of carboplatin and paclitaxel. Interestingly, both *Brip1*^+/*−*^ and *Brip1*^+/+^ mice treated with carboplatin alone exhibited an increased percentage of γH2AX-positive primordial follicles (p < 0.001 and p < 0.0001, respectively) (Fig. 6c,d).

### Co-administration of carboplatin and paclitaxel results in long-term reduced fertility and compromised embryo development in both mutant carriers and wild-type mice

To identify whether the effect of the drugs on mouse fertility was transient or persistent, in vitro* fertilisation* (IVF) experiments were performed three weeks after the last drug/serum injection. The number of oocytes collected were dramatically reduced in *Brip1*^+/*−*^ (p < 0.01) and *Brip1*^+/+^ (p < 0.05) mice treated with the combination of carboplatin and paclitaxel. Interestingly, no oocytes were collected from two combined treated mice, one from each genotype. Additionally, co-administration of carboplatin and paclitaxel affected the fertilisation competence of the oocytes and further embryo development as indicated by the lower fertilisation and blastocyst rates for both *Brip1*^+/*−*^ (p < 0.05 and p < 0.05, respectively) and *Brip1*^+/+^ (p < 0.01 and p < 0.05, respectively) mice (Table 1).

**Table 1:** In vitro fertilisation experiments.

Genotype	Treatment	Oocyte co l lection			Fertilisation and embryo development			
		Mouse ID	# Total	# Degenerated	# 2-cell embryos	% Fertilisation	# Blastocysts	% Blastocysts
Brip1^+/-^	Carboplatin + Paclitaxel	F41	11	0	2	18.2	0	0.0
		F4	0	0	0	0.0	0	0.0
		F1	14	0	4	28.6	2	50.0
		F3	1	0	0	0.0	0	0.0
		F14	14	1	6	46.2	2	33.3
		Summary	40^a^	1	12	30.8 ^b^	4	33.3^b^
	Control	F4	35	0	18	51.4	10	55.6
		F43	43	1	20	47.6	11	55.0
		F4	26	3	12	52.2	8	66.7
		F1	28	5	16	69.6	10	62.5
		F3	26	4	18	81.8	8	44.4
		Summary	158	13	84	57.9	47	56.0
Brip1^+/+^	Carboplatin + Paclitaxel	F3	4	0	0	0.0	0	0.0
		F10	0	0	0	0.0	0	0.0
		F10	16	1	2	13.3	0	0.0
		F31	21	6	1	6.7	0	0.0
		F33	12	0	8	66.7	4	50.0
		Summary	53 ^b^	7	11	23.9^a^	4	36.4^b^
	Control	F44	30	5	16	64.0	8	50.0
		F10	28	4	14	58.3	8	57.1
		F1	17	2	10	66.7	6	60.0
		F41	47	1	28	60.9	18	64.3
		F1	30	5	14	56.0	6	42.9
		Summary	152	17	82	60.7	46	56.1

Oocyte recovery, fertilisation rate and embryo developmental competence in *Brip1*^+*/−*^ and *Brip1*^+*/*+^ mice treated with the combination of carboplatin and paclitaxel or with NaCl 0.9% (control group), three weeks after the completion of the treatment. n = 5/treatment/genotype.

^a^p < 0.01, ^b^p < 0.05.

## Discussion

In this study, we showed that carboplatin and paclitaxel alone have no significant impact on the ovarian reserve of mice while the co-administration of these drugs significantly reduced primordial follicles in the ovaries and impaired fertility potential. The presence of heterozygote mutation of the gene (*Brip1*) involved in DNA repair mechanisms, did not appear to further impact the gonadotoxicity of these drugs. Co-administration of carboplatin and paclitaxel had the same effects on the ovaries of wild type mice both qualitatively and quantitatively. Consequently, the presence of the heterozygous mutation did not appear to have an additional burden on the pool of primordial follicles. Although viable, the homozygous *Brip* mutant mice did not survive the combined treatment. Homozygous mutation of Fanconi anemia genes, including *BRIP1,* was reported in consanguinity family, and was responsible for bone marrow failure and severe congenital malformation^27^. Our results could be particularly concerning for these patients.

To our knowledge, this is the first study evaluating the effects on the ovarian reserve of combined carboplatin and paclitaxel regimen. Despite the fact that the mechanisms of chemotherapy-induced ovarian damage have been extensively studied, our knowledge on the gonadotoxicity of these drugs remains limited^28^. We showed that intraperitoneal administration of carboplatin (50 mg/kg, once a week for three weeks) or paclitaxel (10 mg/kg, twice a week for three weeks) alone did not affect the ovarian reserve. Interestingly, other authors observed a significant reduction of the number of primordial follicles, primary, secondary and antral follicles in the ovaries of mice 28 days after a single intraperitoneal injection of a high dose of carboplatin (80 mg/kg)^29^. In vitro exposure of mouse prepubertal ovaries to carboplatin (5–10 μg/ml) for 24 h also reduced the total follicle number, targeting especially primordial follicles, and has affected the presence of healthy follicle, four days later^30^. The discrepancy between studies could be explained by the differences in the protocol, and suggests that carboplatin has a dose-dependent moderate gonadotoxicity.

The results regarding the gonadotoxicity of paclitaxel are in line with a recent study, assessing the gonadotoxicity of a single high dose of paclitaxel (30 mg/kg) to 7-week-old mice after 1, 6, 11 and 16 days. The authors reported a loss of antral follicles only on day 1 following chemotherapy without affecting primordial, primary or secondary follicular pools^19^. These results confirmed that paclitaxel had a low gonadotoxicity, although one study showed a depletion in the number of primordial follicles only 28 days after a single dose of paclitaxel (7.5 mg/kg)^29^.

Importantly, we showed that the combination of the two drugs dramatically increase follicular damage. Primordial follicle loss following chemotherapy occurs mainly through direct DNA damage of the follicles leading to apoptosis and consequently cell death^31^. In our study, TUNEL analysis did not reveal an increased apoptosis within the treated ovaries at the end of the treatment, as observed in previous studies using alkylating-like agents^32,33^. However, the percentage of primordial follicles with DNA DSBs was significantly higher in mice treated with the combination of carboplatin and paclitaxel, irrespective of the genetic background. Interestingly, similar results were obtained from the mice treated with carboplatin only without affecting the ovarian reserve nor any other parameters. A possible explanation could be that the formation of DNA DSBs induced by carboplatin was transient as the follicles overcame the damage through the repair mechanism. As the results were similar for both *Brip1*^+/*−*^ and *Brip1*^+/+^ mice, it seems that the absence of the one allele did not affect the DNA repair mechanism. Yet, the absence of both *Brip1* alleles was proven to be critical as KO mice did not survive the co-administration of carboplatin and paclitaxel suggesting a strong involvement of the gene in the DNA repair mechanism.

Another mechanism resulting in the depletion of primordial follicles after cytotoxic exposure is the recruitment of primordial follicles by triggering pathways leading to the accelerated activation. It has been reported that cyclophosphamide induces follicular activation in mice, both in vivo and in vitro, through the PI3K pathway^34–36^. The administration of cisplatin and doxorubicin have been also shown to activate the PTEN/Akt/FOXO3 pathway in mice resulting in exhaustion of the ovarian reserve^33,37^. The PI3K/AKT/mTOR pathway is an intrafollicular signalling pathway that has been described as a regulator of physiological and chemotherapy-induced primordial follicular activation^38^. Several growth factors activate the PI3K signalling in the follicles, including KL. Activation of PI3K induces the transformation of phosphatidylinositol 4,5-bisphosphate (PIP2) into phosphatidylinositol 3,4,5-trisphosphate (PIP3). PTEN is a phosphatase that acts as a negative regulator of the PI3K pathway by reversing this transformation, thus downregulation of Pten induces follicle activation. Following the activation of PI3K, the phosphorylation of proteins involved in PI3K signalling increases, leading to the activation of the mammalian target of rapamycin complex 1 (mTORC1) and the phosphorylation of RPS6 (p-RPS6) which contributes to cell growth and primordial follicle activation^39^. In our study, an increased intensity of KL staining was observed in the follicles of *Brip1*^+/*−*^ and *Brip1*^+/+^ mice treated with the combination of carboplatin and paclitaxel. The expression of *Pten* was significantly downregulated in the group of *Brip1*^+/+^ mice treated with the combination of carboplatin and paclitaxel and similar trend was observed for the *Brip1*^+/*−*^ mice. Immunostaining for p-RPS6 showed that the percentage of follicles with phosphorylated RPS6 was increased in both *Brip1*^+/*−*^ and *Brip1*^+/+^ mice in the combined treatment group. Finally, increased Ki67 positive follicles confirmed the cell growth and proliferation. In contrast, no impact of follicular activation was observed in mice treated with carboplatin or paclitaxel alone. This result suggested that the combined administration of the two drugs induced an accelerated primordial follicle activation mediated by the PI3K/AKT/mTOR pathway, resulting in a precocious exhaustion of the ovarian reserve.

Deficiency in BRIP1 gene has been described as the cause for cancer-predisposing Fanconi anemia genes^40,41^. Interestingly, previous studies have demonstrated that mutations in genes involved in the Fanconi pathway also result in early menopause in women and reduced ovarian primordial follicle reserve and reproductive aging in mice^42–44^.

Our data suggested that carrying a *Brip1* gene mutation did not increase the risk of treatment-related ovarian damage but should be confirmed in other mouse models, such as humanized BRCA1 Knock-in mice, as well as in clinical trials. Similarly, we cannot exclude that the effect would be different in other mutation of Fanconi anemia genes. Our study also emphasized the need to evaluate the combination of agents instead of each drug alone. Carboplatin and paclitaxel are also the backbone of epithelial ovarian cancer treatment^45,46^. Although ovarian cancer occurs often later in life, these results could be useful for counselling patients about possible treatment adverse effects, especially when associated with *Brip1* gene mutation which is considered as a low-to-moderate penetrance risk for this cancer for which a risk-reducing salpingo-oophorectomy was recommended in carriers aged between 45 and 50 years^47^.

Cancer treatment in young females is known to be associated with reduced fertility, low rates of pregnancy and live births^48^. For breast cancer survivors, the pregnancy rate is approximately 60% lower than the general population^48^. In our study, we also assessed the efficiency of IVF in both *Brip1*^+/*−*^ and *Brip1*^+/+^ mice treated with the combination of carboplatin and paclitaxel. The experiments were performed three weeks after the completion of the treatment, corresponding to the duration of a complete cycle of folliculogenesis in mouse. The number of oocytes collected was significantly lower as well as the fertilisation and blastocysts formation rates in treated mice compared to untreated mice, indicating a negative impact of the combined treatment on oocyte quality.

This study suggested that co-administration of carboplatin and paclitaxel induces ovarian damage and activates quiescent primordial follicles in mice impairing their ovarian reserve and fertility potential. We acknowledge that the *Brip1* mouse model used in this study cannot be fully extrapolated to *BRCA* mutation carriers. However, this study demonstrates the dramatic cumulative effect of the combined carboplatin and paclitaxel treatment on fertility, regardless of the presence of an heterozygote mutation involved in BRCA1 related DNA repair mechanisms. To conclude, these results highlight the importance to assess the gonadotoxicity of anticancer drugs in preclinical models, and may help clinicians to provide more appropriate information during fertility preservation counselling.

## Methods

### Ethics statement

All experimental procedures were approved by the Animal Ethics Committee of the Medical Faculty at the Université Libre de Bruxelles (683 N, approved on 10/2018 and 07/2020) and were performed in compliance with the European legislation on animal experimentation. The study is reported in accordance with ARRIVE guidelines.

### Animals, treatments and sample collection

C57BL/6 N-Brip1^em1(IMPC)J^/J mice (Jackson Laboratory, ME USA) were housed on a 12 h light/dark cycle with access to food and water ad libitum. Five-to-seven-week-old *Brip1*^*−*/*−*^, *Brip1*^+/*−*^ and *Brip1*^+/+^ female mice (colony established and maintained in house) were allocated into four groups. Treatment doses were based on previous studies^49–52^. The first group received treatment with carboplatin (50 mg/kg, once a week) (ONCO-TAIN 150 mg/15 ml, Hospira, Belgium), the second group with paclitaxel (10 mg/kg, twice a week) (Abraxane 5 mg/ml, Celgene, Belgium), the third group with the combination of carboplatin and paclitaxel (50 mg/kg, once a week and 10 mg/kg, twice a week), and the fourth group with NaCl 0.9% (control group) (n = 5/genotype/group). The drugs were administrated intraperitonially and the duration of each experiment was three weeks.

On the day following the completion of the experiment, mice were anesthetized by administration of anaesthesia (75 mg/kg ketamine (Nimatek, Dechra Regulatory B.V., The Netherlands) and 10 mg/kg xylazine (Rompun, Bayer SA-NV, Belgium)), weighed and blood was collected by cardiac puncture. Blood was centrifuged after coagulation to obtain serum that was stored at − 80 °C before hormonal measurements. Mice were sacrificed by cervical dislocation and the ovaries were collected. The extra surrounding tissue was removed and ovaries were weighed. One ovary was fixed in 4% paraformaldehyde for 24 h at 4 °C, and embedded in paraffin, while the contralateral ovary was placed in RNA*late*r™ Stabilization Solution (Thermo Fisher Scientific, Belgium), and stored at − 80 °C before used for RNA extraction.

### Histology and follicle counts

Paraffin-embedded ovaries were serially sectioned (5 μm). Every fifth section was deparaffinised in toluene, rehydrated with decreasing concentrations of ethanol, and stained with haematoxylin and eosin (H&E) for determining ovarian follicle counts and follicle stage. Only the follicles with a distinct oocyte nucleus were counted and classified as primordial, when an oocyte was surrounded only by flattened granulosa cells, primary, when an oocyte was surrounded by at least one or more cuboidal granulosa cells in a single layer, secondary, when an oocyte was surrounded by two or more layers of cuboidal granulosa cells, and antral, when the antrum was present. Corpora lutea number was assessed every 36th section. All evaluations were performed blindly.

### Electrochemiluminescence immunoassay (ECL)

AMH levels in the blood serum of mice (n = 5/genotype/group) were determined by the automated Elecsys® AMH Plus immunoassay (Roche Diagnostics, Belgium) run on the cobas e 801 analytical unit (Roche Diagnostics) according to the manufacturer’s instructions. Analysis was performed by the LUHB laboratory (Brussels academic hospital laboratory).

### Immunostaining

Immunohistochemistry and immunofluorescence were performed to evaluate DNA damage by γH2AX and the follicular activation process, including Ki-67, KIT LIGAND and p-RPS6. Ovarian tissue sections were deparaffinised in toluene and rehydrated with decreasing concentrations of ethanol. Heat-mediated antigen retrieval was performed in citrate buffer (ScyTek Laboratories, Belgium) and endogenous peroxidases were quenched for 30 min in 1% hydrogen peroxide (Merck Millipore, Belgium). Non-specific binding of antibodies was blocked with 5% normal goat serum (Vector Laboratories, Belgium). Sections were incubated with primary antibody against Phospho-Histone (γ)H2AX (Ser139) (1:100; Cell Signaling Technology #9718S), Ki-67 (1:400; BD Bioscience, Belgium #556003), KIT LIGAND (1:100; Abcam, #64677) and p-RPS6 (1:100; Cell Signaling Technology #2217S OR 4858) overnight at 4 °C in a humidify environment followed by incubation with the appropriate secondary antibody. For γH2AX, Ki-67 and p-RPS6 immunostaining, the signal was amplified using an ABC Vectastain Elite kit (Vector Laboratories), revealed with a diaminobenzidine peroxidase substrate kit (Vector Laboratories) and then counterstained with haematoxylin. For KIT LIGAND, slides were incubated with conjugated avidin fluorochrome fluorescein (1:200; Vector Laboratories, #A2001). Tissue sections stained with the primary antibody omitted were used as negative controls. Slides were examined using a Leica DM 2000 fluorescence microscope. Image analysis was performed using Zen 2.3 (Carl Zeiss, Germany). Quantification was performed on at least 3 randomly selected sections per ovary per mouse. For γH2AX expression, follicles with stained nucleus were counted as positive and reported as a proportion (%) of the total follicle number per ovary. For Ki-67 expression, follicles with at least one granulosa cell stained were counted as positive and reported as a proportion (%) of the total follicle number per ovary. For p-RPS6 expression, follicles with stained cytoplasm of oocytes or granulosa cells were counted as positive and reported as a proportion (%) of the total follicle number per ovary. For the quantification of KIT LIGAND staining, at least 20 randomly selected primordial and primary follicles per ovary per mouse were counted. The ratio of the mean fluorescein signal intensity to the mean Hoechst signal of the same follicle area was reported (%).

### TUNEL assay

Global apoptosis was assessed by TUNEL staining (In Situ Death Cell Detection Kit, Roche). Tissue sections were deparaffinised, rehydrated and permeabilized with proteinase K (20 μg/ml in 10 mM Tris pH 7.4, Qiagen, The Netherlands). After labelling with TUNEL reagents according to manufacturer’s instructions, sections were counterstained with Hoechst (1 μg/ml). Sections treated with 50 IU/ml DNAse I (Invitrogen, Belgium) served as positive control, while for the negative control the enzyme was replaced with phosphate buffer solution. The sections were observed under a Leica DM 2000 fluorescent microscope and the image analysis was performed using ZEN 2.3 software (Carl Zeiss). At least 3 randomly selected sections per ovary per mouse were assessed. TUNEL-positive (red) pixels and Hoechst-positive (blue) pixels were quantified and the degree of global apoptosis was expressed as the percentage of the TUNEL-positive counts to the total Hoechst-positive counts per ovary.

### Gene expression quantification by QPCR

The expression of gene of interest was assessed by QPCR. Total RNA was extracted from one ovary per mouse (n = 5/genotype/group) using the ReliaPrep™ RNA Tissue Miniprep System (Promega, The Netherlands). The quantity and purity of RNA samples was determined using a NanoDrop spectrophotometer (Thermo Fisher Scientific, Belgium). Reverse transcription reactions were preformed using 200 ng of total RNA and random primers. For each QPCR reaction, 2 ng of cDNA were mixed with SYBR® Green Master mix (Applied Biosystems, Belgium) and 10 μM of the targeted genes forward and reverse primers (Eurogentec, Belgium) (Supplementary Table S1). Each sample was analysed in triplicate and the gene RPL19 was used as housekeeping gene. The expression level of each gene was calculated by the comparative Ct method (ΔΔCt) and the fold change was calculated by the equation 2^−ΔΔCt^.

### In vitro fertilisation (IVF)

The effect of the drugs on mouse fertility was evaluated by IVF. Five-to-seven-week-old *Brip1*^+/*−*^ and *Brip1*^+/+^ female mice were allocated into two groups and treated as previously described. The first group received treatment with the combination of carboplatin and paclitaxel (50 mg/kg, once a week and 10 mg/kg, twice a week, respectively), and the second group with NaCl 0.9% (control group) (n = 5/genotype/group) for a total of three weeks. Three weeks after the completion of the treatment, mice received an intraperitoneal injection of pregnant mare serum gonadotropin (5 IU/Bioworld, bioPlus Chemicals 1000 IU) followed by human chorionic gonadotropin injection (5 IU/Chorulon 1500 IU, Intervet International, Brussels) 48 h later to induce follicular development and ovulation. On the day of IVF, freshly harvested sperm from three-to-six-month-old *Brip1*^+/+^ adult male mice was allowed for capacitation in pre-warmed (HTF, EmbryoMax HTF, Millipore, Germany-3%BSA + 10 μl/ml NEAA) for 60 min (37 °C, 5% CO_2_). Female mice were sacrificed and the oviducts were dissected. Through a tear at the ampulla, cumulus oocyte complexes (COCs) were collected in prewarmed HTF and incubated for 30 min before 5 μL of sperm was added. Four hours later, COCs were washed to remove the excess sperm and the fertilised oocytes were placed in a drop of KSOM media (company EmbryoMax, Millipore, Germany) and incubated overnight. Embryo development was observed from the two-cell cleavage to blastocyst stage.

### Statistical analysis

Data were analysed using GraphPad Prism 8 Software (La Jolla, USA). To determine the effect of treatment, normally distributed data were analysed by one-way ANOVA followed by Dunnett’s post hoc test. For data that were not normally distributed, Kruskal–Wallis followed by Dunn’s post hoc test was performed. To determine statistical significance between groups of mice of different genotype student’s t-test or Mann–Whitney were performed. All results are given as mean ± SD and differences were considered significant when p < 0.05.

## Supplementary Information

Supplementary Information.
