# Supplementary material for: Ovarian toxicity of carboplatin and paclitaxel in mouse carriers of mutation in BRIP1 tumor suppressor gene
Source: Sci Rep. 2022 Feb 1;12:1658. doi: 10.1038/s41598-022-05357-x (PMC8807594; doi:10.1038/s41598-022-05357-x)
Supplement: Supplementary file 1 — Supplementary Information. [file 41598_2022_5357_MOESM1_ESM.pdf]

**Table S1.** Gene Primers Sequences

| Gene Symbol | Official Name | Primers' Sequence (Forward-Reverse) |
|---|---|---|
| PTEN | phosphatase and tensin homolog | F: TTC-ACG-TCC-TAC-CCC-TTT-GC |
| | | R: GGT-CCA-GAG-CCC-AGG-TAG-AA |
| RPL19 | ribosomal protein L19 | F: GAA-AGG-TGC-TTC-CGA-TTC-CA |
| | | R: TGA-TCG-CTT-GAT-GCA-AAT-CC |
